# Maxillary protraction and vertical control utilizing skeletal anchorage for midfacial-maxillary deficiency

**DOI:** 10.1590/2177-6709.26.6.e2120114.oar

**Published:** 2021-12-15

**Authors:** Kensuke MATSUMOTO, Nipul TANNA

**Affiliations:** 1 University of Pennsylvania, School of Dental Medicine, Department of Periodontics and Orthodontics (Philadelphia/PA, USA).; 2 University of Pennsylvania, School of Dental Medicine, Department of Orthodontics (Philadelphia/PA, USA).

**Keywords:** TADs, Palatal expander, Facemask, Class III correction, Skeletal anchorage

## Abstract

**Introduction:**

The efficacy and efficiency of early treatment of skeletal Class III patients with facemask therapy are well-documented; however, very few cases for adolescents or adults were reported.

**Objective:**

The aim of this case report was to demonstrate skeletal and dental correction of a post-pubertal-growth-spurt patient whose malocclusion consisted of a skeletal Class III with slight transverse deficiency, a high mandibular plane angle, and a retrusive maxillary complex.

**Case report:**

A 13-year-5-months old Hispanic female was diagnosed as a retrognathic maxilla and mandible, a high mandibular plane angle, open bite pattern, a bilateral Angle Class I molar relationship with an anterior crossbite on the maxillary lateral incisors. A TAD-supported Haas rapid palatal expander was utilized for maxillary protraction combined with a facemask, vertical control, and maxillary molar distalization with fixed appliance.

**Results:**

The total treatment time was 26 months. An improved facial profile with maxillary lip support and more prominent cheeks was established. Adequate vertical control prevented a change in the mandibular plane angle even though facemask treatment can increase the vertical dimension. After the 18-month retention, excellent stability of the treatment results was shown.

**Conclusion:**

With skeletal anchorage and facemask treatment, orthodontists have the ability of expanding and protracting the maxilla without tipping maxillary molars buccally and without the risk of unfavorable periodontal consequences. A TAD-supported Haas rapid palatal expander allowed to control the vertical dimension and distalize molars, while minimizing undesired consequences.

## RESUMO

**Introdução:**

A eficácia e a eficiência do tratamento precoce de pacientes Classe III esquelética usando máscara facial são bem documentadas na literatura. Entretanto, poucos casos de adolescentes ou adultos foram descritos.

**Objetivo:**

O objetivo do presente relato de caso foi demonstrar as correções esqueléticas e dentárias em uma paciente pós-surto de crescimento puberal, cuja má oclusão consistia de uma Classe III esquelética com deficiência transversa suave, plano do ângulo mandibular aumentado e complexo maxilar retruído.

**Relato de caso:**

Uma paciente hispânica, com treze anos e cinco meses de idade, foi diagnosticada com retrusão maxilomandibular, ângulo do plano mandibular aumentado, padrão de mordida aberta, relação bilateral de molares em Classe I e mordida cruzada nos incisivos laterais superiores. Um aparelho de Haas suportado por dispositivos de ancoragem temporária (DATs) foi usado para a protração maxilar, combinado com máscara facial, controle vertical e distalização de molares superiores com aparelho fixo.

**Resultados:**

O tempo total de tratamento foi de 26 meses. Houve melhora no perfil facial, com aumento no suporte labial e bochechas mais proeminentes. O controle vertical adequado evitou uma mudança no plano do ângulo mandibular, apesar de o tratamento com máscara facial poder aumentar a dimensão vertical. Após contenção por 18 meses, uma excelente estabilidade do tratamento foi observada.

**Conclusão:**

Com a ancoragem esquelética e o tratamento com máscara facial, os ortodontistas têm a possibilidade de expandir e protrair a maxila sem a inclinação vestibular dos molares superiores e sem o risco de consequências periodontais desfavoráveis. Um expansor de Haas suportado por DATs permitiu o controle da dimensão vertical e distalização dos molares, com redução de consequências indesejáveis.

## INTRODUCTION

The efficacy and efficiency of skeletal Class III patients in early treatment are well-documented. Maxillary deficiency is often treated with maxillary protraction, and may be with or without palatal expansion. Treatment should be carried out in patients less than 10 years of age to enhance the orthopedic effect.^1^ ^,^ ^2^ However, there are some reports in the literature that there is no statistically significant difference between younger and older (> 10-year-old) children.^3^ ^-^ ^6^ Discrepancy between the skeletal and chronological ages may be a factor, and it might be better to consider the skeletal age as a clinical indicator to determine the effectiveness of using a facemask.^7^ However, even if correction can be achieved in all growing patients, the skeletal changes may be smaller in older children. This case report demonstrates the efficacy of a TAD-supported Haas rapid palatal expander in conjunction with a facemask utilized for transverse correction, sagittal correction, and vertical control. 

### DIAGNOSIS AND ETIOLOGY

A 13-year-5-months old Hispanic female presented with the following chief complaint: *“I don’t like my front teeth, which are not straight”*. Her medical history was noncontributory, and she was in post menarche. She had routine hygiene visits every six months and was stable from periodontal and restorative perspectives. Her oral hygiene was fair. 

**Figure 1:**
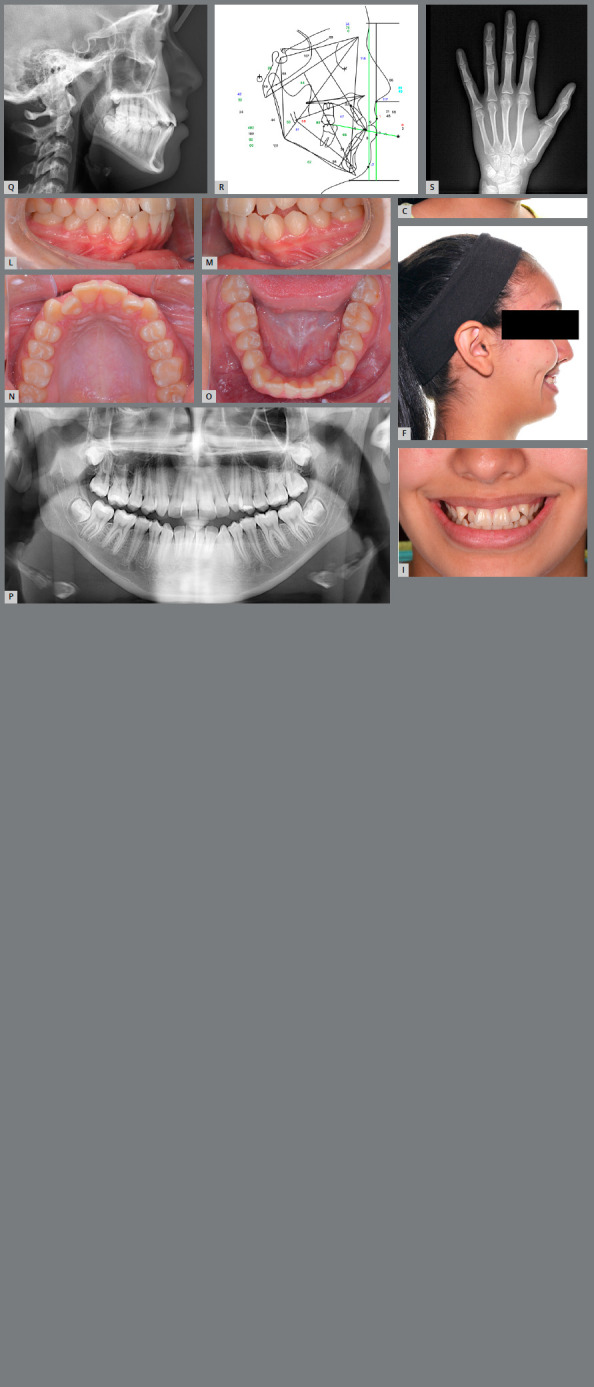
A-I) Pretreatment extraoral photographs.

Her nasal dorsum was deviated slightly toward the right side (Figs 1A-I). She had a straight profile, with a dolichocephalic facial-type, an obtuse nasolabial angle, a retrusive upper lip, and a flat chin. Facial thirds were well balanced. Her cheeks were flattened, and the maxillary complex appeared retrusive. She had a symmetrical face and competent lips at rest, with a thin upper lip. The smile arc was inconsonant, with a 90% incisor display.

Intraorally, she had bilateral Angle Class I molar relationships, with an anterior crossbite on the maxillary lateral incisors (Figs 1J-O). The left maxillary canine was insufficiently erupted. Her maxillary molars were buccally inclined, and mandibular molars were lingually inclined. After correcting their inclinations, molar relationships would be of bilateral crossbite. Therefore, her transverse skeletal diagnosis was of maxillary transverse deficiency. Her maxillary and mandibular midlines were deviated 1 mm toward the left, with an overjet of 1.6 mm and overbite of 0.5 mm. There was crowding of 5 mm in the maxillary arch and 5 mm in the mandibular arch. Pretreatment panoramic radiograph showed partial development of all third molars (Fig 1P).

The pretreatment lateral cephalometric radiograph and tracing (Figs 1Q, 1R) and analysis (Table 1) demonstrated a Class III skeletal pattern (ANB = 0.5?, Wits appraisal = -5.2 mm), with a retrognathic maxilla (SNA = 74.2?) and mandible (SNB = 73.7?). The SN.MP angle of 42.4? and the FMA of 33.6? reflected a high mandibular plane angle and open bite pattern. The maxillary incisors were proclined (U1.SN = 107.2?). The mandibular incisors were proclined (IMPA = 97.5?) and procumbent (L1.NB = 7.6 mm). 

**Table 1:** Cephalometric measurements at the stages of treatment and retention.

Measurement	Norm	Pretreatment (Pre-tx)	Post-RPE with facemask	Post-treatment (Post-tx)	Retention	Change (Pre-tx and post-RPE)	Change (Pre-tx and post-tx)	Change (Post-tx and retention)
Skeletal								
SNA (degrees)	81.6	74.2	75.8	75.9	75.6	1.6	1.7	-0.3
SNB (degrees)	78.6	73.7	72.4	72.9	72.3	-1.3	-0.8	-0.6
ANB (degrees)	2.9	0.5	3.4	3.2	3.2	2.9	2.5	0.2
Wits (mm)	1.1	-5.2	0.8	-1.5	-1.5	6.0	3.5	0.2
SN.GoGn (degrees)	31.8	42.4	44.2	45.4	45.4	1.8	0.2	2.8
FMA (degrees)	20.6	32.2	33.4	34.3	34.3	1.2	.03	1.8
Dental								
U1.SN (degrees)	104.0	107.2	104.8	101.2	101.2	-2.4	-4.4	-1.6
U1.NA (degrees)	22.7	33.0	29.0	25.6	25.6	-4.0	-6.9	-0.5
U1-NA (mm)	4.3	8.5	6.7	6.0	6.0	-1.8	-0.9	-1.6
L1.NB (degrees)	29.1	33.6	29.1	32.6	32.6	-4.5	-4.1	3.1
L1-NB (mm)	6.6	7.6	6.9	8.3	8.3	-0.7	1.4	-0.7
IMPA (degrees)	98.0	97.5	92.6	94.9	94.9	-4.9	-3.5	0.9
Soft tissue								
Nasolabial angle (degrees)	105.0	117.2	113.5	105.6	111.5	-3.7	-11.6	5.9
L lip to E-plane (mm)	0.0	-0.7	-0.5	2.7	1.3	0.2	3.4	-1.4

The growth potential was evaluated, and cervical vertebrae maturation stage^8^ demonstrated CS4, which indicated that her peak mandibular growth occurred within 1-2 years before this stage. Radiographic evaluation of skeletal maturation with the hand-wrist film^9^ showed the ulna and the radial epiphyses were fused (skeletal maturation indicator = SMI 11), and her skeletal age was 16 years old (Fig 1S). 

The etiology of her malocclusion may have been a combination of genetic and developmental factors. She had a skeletal Class III with bilateral Angle Class I molar relationships. Hence, the possible explanation would be early loss of maxillary deciduous dentition.

### TREATMENT OBJECTIVES

The treatment objectives were: (1) to increase facial convexity, with maxillary protraction; (2) to minimize the increase of the mandibular plane angle, with vertical control; (3) to increase incisor display; (4) to maintain broad arch form, to create a more balanced esthetic face; (5) to distalize maxillary molars; and (6) to maintain mandibular molars, to achieve Class I molar relationship. 

### TREATMENT ALTERNATIVES

Three treatment options were considered.

1. Extraction of four first premolars, to align the maxillary lateral incisors, retract mandibular incisors, and close the remaining spaces reciprocally, to achieve anterior coupling and a Class I canine relationship. This treatment would provide the solution of the arch-length deficiency and possibly a stable tooth alignment. However, this option would not improve the retrusive maxillary complex, and could even worsen the profile.
2. Extensive interproximal reduction (IPR) on maxillary and mandibular anterior teeth, to relieve crowding. The disadvantage of this option would be proclination of both maxillary and mandibular incisors, and maintenance of the retrusive maxilla. Hence, the facial and smile esthetics would not be optimized.
3. Maxillary expansion with a TAD-supported Haas rapid palatal expander (TAD-Haas RPE) and protraction with a facemask. Distalization of maxillary posterior teeth with the TADs would also provide predictable vertical control. Since she was at post-pubertal growth spurt, being a skeletally mature patient, conventional RPE and/or facemask treatment would provide more dental and less skeletal correction. The orthopedic effects of TAD-Haas RPE and facemask treatment would allow the maxilla to come forward and downward, while minimizing negative dental compensation. This treatment option would enhance both the profile and smile esthetics, by increasing incisal display. However, the patient’s compliance would be the key for this treatment option.

The patient and her parents rejected the options of extraction and extensive IPR. The third option, TAD-Haas RPE with a facemask, was accepted because of the optimal facial and smile esthetics without tooth extraction. 

### TREATMENT PROGRESS

**Figure 2:**
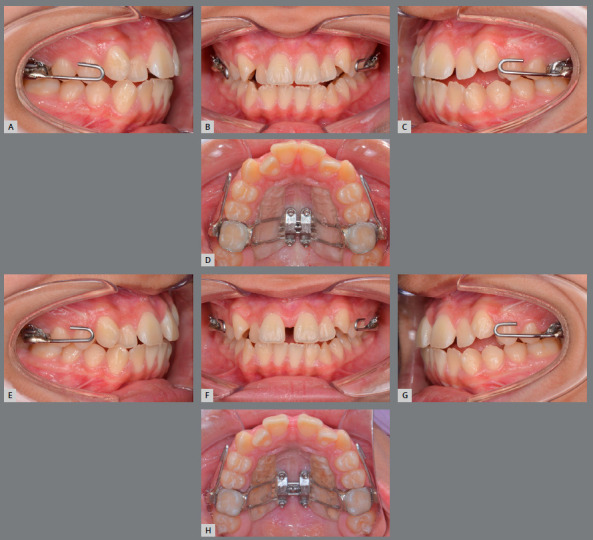
A-D) TAD-RPE with acrylic coverage and facemask hooks on the maxillary first molars: Frontal, lateral and occlusal views before expansion. E-H) After 21 days of expansion.

**Figure 3:**
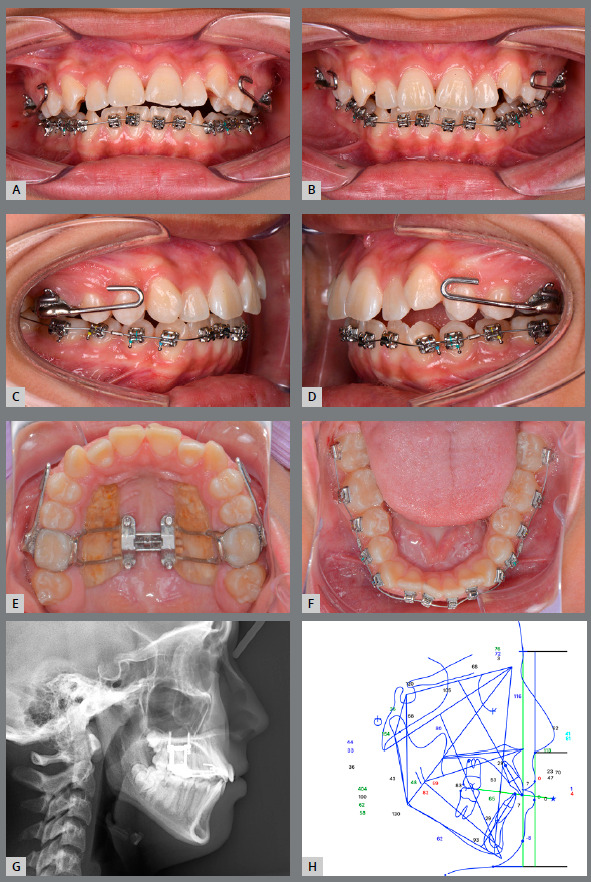
Intraoral photographs **(A-**F), lateral cephalometric radiograph **(**G), and tracing **(**H) at completion of TAD-Haas RPE and facemask treatment.

**Figure 4:**
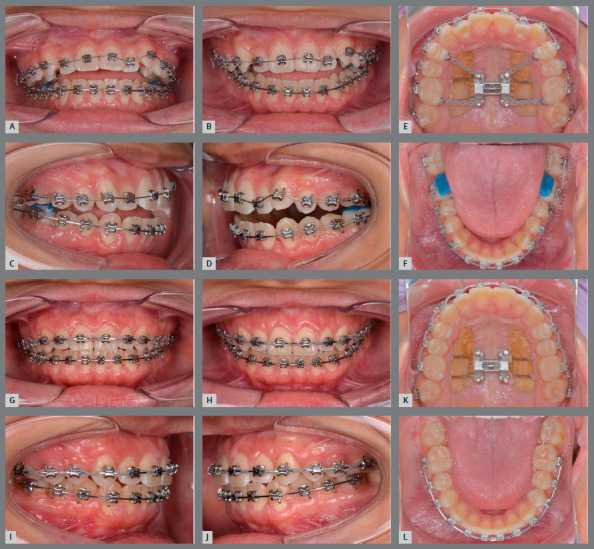
A-F) Molar bands were removed and mandibular leveling and alignment was completed. G-L) Completion of maxillary molar distalization.

A TAD-Haas RPE, consisting of acrylic palatal coverage and bands attached to the maxillary first molars, was applied in order to minimize the buccal tipping of the alveolar bone and the molar axes (Figs 2A-D). Additionally, facemask hooks were soldered to the bands. The expander was activated by turning the jackscrew once a day for 32 days, and the facemask was initiated simultaneously. Elastics were connected to the outer bow of the facemask in a 30? downward and forward direction, delivering 500 g of force per side for 13 to 14 hours per day, for 6 months (Figs 2E-H). The expansion resulted in 8 mm at the jackscrew. After the expansion was completed, the mandibular arch was bonded with self-ligating brackets (0.022-in preadjusted appliances, Roth prescription) (Figs 3A-F). A lateral cephalometric radiograph was taken after TAD-Haas RPE and facemask treatment (Fig 3G). Cephalometric tracing of post-TAD-Haas RPE and facemask treatment (Fig 3H) showed the maxilla protracted forward and downward, and there was clockwise rotation of the mandible. Both maxillary and mandibular incisors retroclined, and there was minimum movement of both maxillary and mandibular molars. Leveling and alignment was started with 0.014-in Nitinol, and progressed to 0.019 x 0.025-in stainless steel archwires. After completion of the facemask treatment, only molar bands of the TAD-Haas RPE were removed and converted to molar brackets, but the RPE was maintained, the maxillary arch was bonded, and the leveling and alignment phase was initiated. Palatal attachments on maxillary first premolars and first molars were connected with the TADs, to increase anchorage and control the vertical dimension (Figs 4A-F). The maxillary distalization for Class II correction was initiated bilaterally, with open coils between second premolar and first molar. Then, sequential distalization was accomplished (Figs 4G-L). Once anterior teeth were coupled, the TAD-Haas RPE was removed. Finishing and detailing was completed. The patient was debonded and retained with a maxillary Hawley wraparound and fixed mandibular canine to canine retainer. Treatment was completed in 26 months. 

### TREATMENT RESULTS

**Figure 5:**
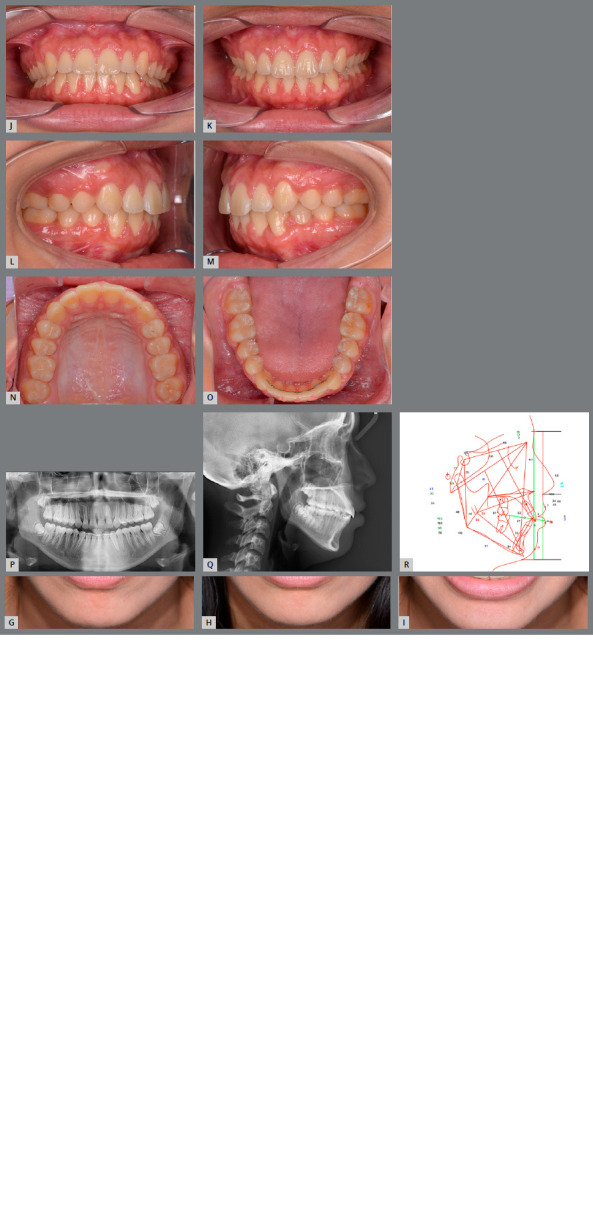
A-I) Post-treatment extraoral photographs.

**Figure 6:**
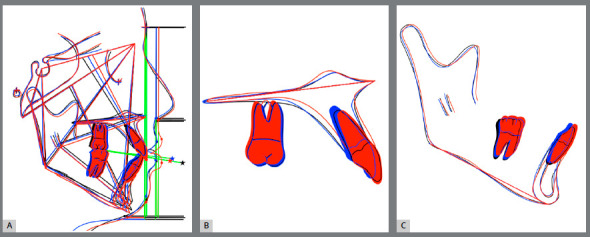
Pretreatment (black), post-TAD-Haas RPE and facemask treatment (blue) and post-treatment (red) cephalometric tracings superimpositions: **A**) superimposed on the sella-nasion plane at sella; **B**) superimposed on the palatal plane at ANS; **C**) superimposed on the mandibular plane at menton.

The facial profile was enhanced, with maxillary lip support and nasolabial angle reduction, utilizing maxillary protraction (Figs 5A-I). Flattened cheeks became more prominent. The smile esthetics was enhanced, with optimal anterior tooth display, adequate gingival display, and consonant smile arc. Ideal anterior coupling, midline correction, and Class I canine and molar relationships were achieved (Figs 5J-O). The post-treatment panoramic radiograph showed excellent root parallelism and minimum root resorption (Fig 5P). The post-treatment lateral cephalometric radiograph, tracing and the superimpositions exhibited maxillary downward and forward movement (Figs 5Q-R, Figs 6A-C). The changes in SNA (+2.1?), ANB (+2.3?), and Wits appraisal (+3.0 mm) demonstrated an improvement of the skeletal Class III. The maxillary incisors were retroclined, retracted, and extruded. Mandibular incisors were retroclined and extruded. Maxillary first molars were slightly distalized, and there was no vertical change. 

**Figure 7:**
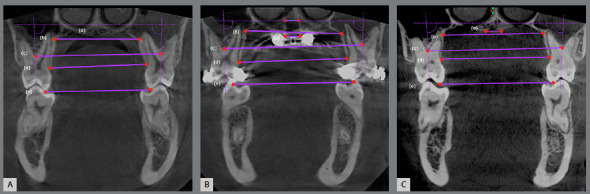
Measurements at pretreatment **(A)**, post-expansion **(B**), and post-treatment **(C**). a = suture (mm), b = U6 basal bones (mm), c = U6 furcations (mm), d = U6 CEJs (mm), and e = U6 palatal cusps (mm).

Mandibular first molars were extruded. Adequate vertical control prevented a change in the mandibular plane angle even though facemask treatment can increase the vertical dimension (Table 1). Cone Beam Computed Tomography (CBCT)images at post-expansion confirmed a 5.7-mm skeletal expansion at the level of the first molars, which was maintained after the orthodontic treatment (Figs 7A-C, Table 2). 

**Table 2:** Measurements in the transverse dimension at pretreatment, post-expansion and post-treatment.

Jackscrew 8mm expansion	Pretreatment (A)	Post-expansion (B)	Post-treatment (C)	Differences			Percentage of jackscrew
				B-A	C-B	C-A	B-A
Suture (mm)	0.00	5.68	5.66	5.68	-0.02	5.66	71.0%
U6 basal bones (mm)	31.59	36.64	36.56	5.05	-0.08	4.97	63.1%
U6 furcations (mm)	46.69	52.43	49.79	5.74	-2.64	3.10	71.8%
U6 CEJs (mm)	35.89	40.82	40.08	4.93	-0.74	4.19	61.6%
U6 palatal cusps (mm)	39.25	46.00	41.77	6.75	-4.23	2.52	84.4%
UR6 inclination (degrees)	87.0	89.0	87.0	2.0	-2.0	0.0	-
UL6 inclination (degrees)	88.0	88.0	87.0	0.0	-1.0	-1.0	-
UR alveolar bone inclination (degrees)	101.0	101.0	98.5	0.0	-2.5	-2.5	-
UL alveolar bone inclination (degrees)	106.0	102.0	105.0	-4.0	3.0	-1.0	-

**Figure 8:**
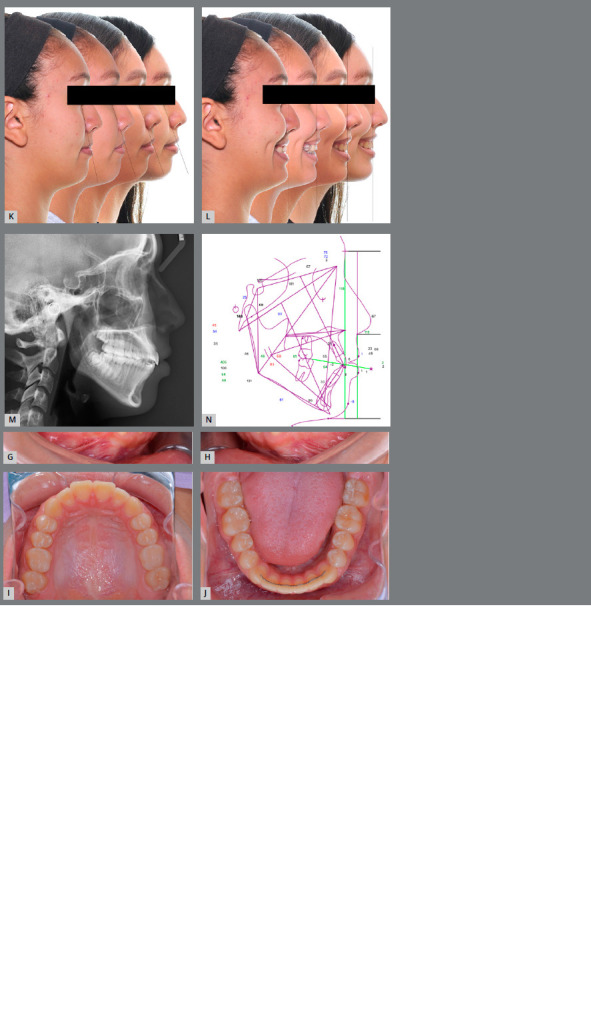
Extraoral **(A-**D) and intraoral photographs **(E-J**) at 18-month retention.

At the 18-month retention visit, excellent stability of the treatment results was shown (Figs 8A-D). Patient’s profile has been maintained and there was optimal lip projection. Angle Class I molar relationship, anterior overjet and overbite were maintained (Figs 8E-J). Figures 8K and 8L depict the profile changes through the treatment. The facial profile was improved, with maxillary lip support and improved nasolabial angle, and more prominent cheeks. The lateral cephalometric radiograph and tracing of post-treatment and 18-month retention (Figs 8M and 8N) showed the maxilla was stable, mandible presented backward rotation, and maxillary incisors were tipped back slightly. Mandibular dentition was retained. 

## DISCUSSION

Systematic reviews and meta-analyses have shown that the orthopedic effect produced by maxillary protraction allows the maxilla to move forward and downward. Additionally, there is mandibular downward and backward rotation, along with dental effects.^2^ ^,^ ^10^ ^,^ ^11^ The efficacy of facemask treatment for skeletal Class III patients has been discussed regarding the timing, treatment duration, impact of the combination with RPE, and usage of skeletal anchorage devices. Facemask treatment is effective when patients starting the treatment are younger or older children.^6^ ^,^ ^12^ However, after 10 years of age, decrease of the skeletal changes, increase of dental compensation, and longer treatment time were reported.^1^ ^-^ ^5^ ^,^ ^11^ ^,^ ^12^ Even though skeletal Class III correction might be achieved in all age groups (3-12 years old), treatment should be started as soon as the diagnosis is made, because younger patients showed greater and faster results in less time.^12^ In the present article, the patient was a 13-year-5-months-old female in post menarche, CS4 (CVS) and 16-year-old skeletal age, according to a hand-wrist film. Her growth spurt has passed, and she is skeletally mature. At this age, facemask treatment alone would not provide the skeletal improvements, but most likely would have dental effects. 

CBCT images at post-expansion confirmed the skeletal expansion in the midpalatal suture at the level of the first molars was 5.7 mm, which was 71% of the jackscrew expansion; the first molars tipped 2.0? buccally, and alveolar bone inclination changed 4.0? palatally (Figs 3A-F and Table 2). Garrett et al.^13^ reported 38% of skeletal expansion in the first molar region with the Hyrax RPE in patients (mean age = 13.8 years). A mean of 52.82% (4.33 mm) midpalatal suture opening at the first molars was obtained in children (mean age = 9.9 years) who were treated with a bonded RPE.^14^ Lin et al.^15^ evaluated the immediate effects of RPE on the transverse skeletal and dentoalveolar changes with bone-borne expander (TAD-RPE) and tooth-borne expander (Hyrax expander) in late female adolescents. They reported the Hyrax group produced more buccal tooth tipping (13.1? vs 2.3?), more buccal alveolar bone bending (7.3? vs 2.1?), and less skeletal expansion (1.14 mm vs 1.99 mm) at the maxillary-first-molar level. Additionally, they found that significant buccal dehiscence occurred in the Hyrax group. 

In summary, bone- or tissue-borne expanders produced greater orthopedic effects and fewer dentoalveolar side effects, compared to the tooth-borne expanders. TAD-Haas RPE showed excellent results for maximizing the skeletal changes and minimizing the dentoalveolar compensations. As a long-term stability after expansion, RPE treatment did not influence the sagittal position of the apical jaw bases or the facial vertical dimension.^16^

RPE treatment alone has shown that there is downward and forward movement of the maxilla.^17^ ^-^ ^19^ The mean SNA, ANB, mandibular plane angle (MP.SN) increased by 0.35?, 1.00?, 1.72?, respectively.^19^ As dental changes, average decreases of the U1.SN and IMPA were reported as 0.43? and 0.59?, respectively.^19^ Therefore, RPE can be beneficial in skeletal Class III patients for promoting maxillary forward movement and incisal uprighting. 

Regarding the facemask in combination with RPE, some studies support that combination treatment is better than facemask alone,^20^ ^-^ ^22^ while others advocated that there were no significant differences between expansion and non-expansion groups with facemask therapy under the age of 10 years.^23^ ^-^ ^25^ In a systematic review,^2^ the treatment duration was much longer in the non-expansion group. More skeletal effect and less dental change were produced with the RPE, whereas more dental change was produced with the non-expansion appliance. Additionally, Kim et al.^2^ speculated that the more skeletal effects and less dental changes might be expected if the RPE with a palatal acrylic was utilized to enhance the anchorage. Hence, in the preset case, it was decided to combine a palatal acrylic support to the RPE. 

Currently, facemask with TAD-RPE,^26^ ^-^ ^29^ or direct bone-anchored maxillary protraction devices (BAMP)^22^ are available. Utilizing skeletal anchorage devices effectively allows for maxillary protraction, reduces dental compensation, and maximizes skeletal changes. Treatment of 16 growing Class III patients (mean age 9.5 ± 1.3 years) utilizing a hybrid Hyrax-facemask combination was evaluated and there was a significant improvement: SNA = +2.0?; SNB = −1.2?; ANB = +3.2?; Wits appraisal = +4.1 mm, with minimum change in vertical skeletal relationships and maxillary incisor inclination.^26^ Ngan et al.^29^ reported that patients (mean age 9.8 years) who were treated with the tooth-borne protraction had changes of SNA = +0.69?, SNB = -1.73?, ANB = +2.42?, Wits = +2.52mm, U1-SN = +2.19?, and IMPA = - 4.99?, while the bone-borne protraction group (mean age 9.6 years) had changes of SNA = +1.59?, SNB = -0.80?, ANB = +2.40?, Wits = +2.58mm, U1-SN = -2.03?, and IMPA = -1.67?. Therefore, the tooth-borne group had more maxillary incisor proclination, due to mesialization of posterior teeth with dental compensation, while the bone-borne group had less downward movement of the A-point, less mandibular plane opening, and more maxillary incisor eruption. Regarding the locations of TADs placement, anterior palate provides more bone thickness. The midpalatal area within 1 mm of the midsagittal suture had the thickest bone available in the whole palate, and the thickness of bone tended to decrease laterally and posteriorly.^30^ Maximum effective bone heights were detected within a T-shaped area at the midpoint of first premolars and at contact point first-second premolars.^31^ However, both researches mentioned that high interindividual variation was found and should be carefully considered by the clinician. Although successful results with BAMP in the late mixed or permanent dentition age of 10-14 years were reported,^32^ this option is less frequently used, due to the requirement of the surgeries for placement and removal of the miniplates. 

Considering all factors in this case report, TAD-Haas RPE with facemask treatment was utilized in order to maximize the skeletal changes, minimize the dental effects, and shorten the treatment duration. As the results of 5.7-mm maxillary skeletal expansion and protraction for six months, the changes of SNA, SNB, ANB, Wits, SN.GoGn, U1.SN, and IMPA were +1.6?, -1.3?, +2.9?, +6.0 mm, +1.8?, -2.4?,+1.6?, and -4.9?, respectively. The results for this post-growth-spurt patient were compatible or even preferable to a previous study^29^ in which 10-year-old children were treated with TAD-RPE and facemask. 

Once the maxillary expansion and overcorrection of the maxillary protraction to an end-to-end Class II molar relationship was completed, the TADs for the RPE were utilized for controlling the vertical dimension and bilateral maxillary molar distalization (Figs 4A-L). During the phase II treatment, the TADs were connected with the attachments on the maxillary first premolars and molars, for controlling the posterior vertical dimension and increasing the anchorage against the molar distalization. According to a recent systematic review regarding the effects of TAD-supported maxillary molar distalization in Class II malocclusions,^33^ the mean molar distalization values varied from 1.8 mm to 6.4 mm. In the present patient, 2-mm distalization was accomplished with simple modification of the TAD-Haas RPE (Fig 6B).

After 18-month retention, photographs and cephalometric tracing (Figs 8A-D, 8N and Table 1) showed a stable result of the maxillary protraction, mesialized maxillary molars, and uprighted maxillary incisors. 

## CONCLUSION

With skeletal anchorage and facemask treatment, orthodontists have the ability of expanding and protracting the maxilla without tipping maxillary molars buccally and without the risk of unfavorable periodontal consequences. A TAD-Haas RPE allowed to distalize molars and control the vertical dimension, while minimizing undesired consequences. 
